# The Effects of Citric Acid Crosslinking on Fabrication and Characterization of Gelatin/Curcumin-Based Electrospun Antioxidant Nanofibers

**DOI:** 10.3390/antiox12071387

**Published:** 2023-07-05

**Authors:** Reem Hasan, Gulum Sumnu, Serpil Sahin, Emel Oz, Fatih Oz

**Affiliations:** 1 Department of Food Engineering, Middle East Technical University, Ankara 06800, Türkiye; reem.hasan@metu.edu.tr (R.H.); serp@metu.edu.tr (S.S.); 2 Department of Food Engineering, Faculty of Agriculture, Ataturk University, Erzurum 25240, Türkiye; emel.oz@atauni.edu.tr (E.O.); fatihoz@atauni.edu.tr (F.O.)

**Keywords:** electrospinning, crosslinking, curcumin, phenolics, nanofibers, sustainability

## Abstract

Nanofibers, produced through the novel method of electrospinning, have a high ratio of surface area to volume, which allows them to have different optical, electrical, thermal, and mechanical properties than macroscale materials. In this study, it was aimed to produce nanofibers with gelatin and curcumin. The effects of gelatin concentration and crosslinking with citric acid on the characteristics of electrospun nanofibers were studied. Gelatin film containing neither citric acid nor curcumin was used as control. Solutions were evaluated by solution conductivity, color analysis, and rheological properties. Obtained nanofibers were characterized by morphological analysis (SEM), antioxidant activity (AA), thermal properties (TGA, XRD, DSC), water vapor permeability (WVP), and Fourier transform infrared (FTIR) analysis. It was found that the functional groups of gelatin were not changed significantly but some degree of crosslinking was seen, as indicated by the changes in AA, crystallinity, etc. Improvement in antioxidant activities was seen, which was the highest for gelatin and curcumin films (32%). The highest melting temperature (78 °C) and WVP (2.365 × 10^−10^ gm^−1^ s^−1^ Pa^−1^) was seen for gelatin and curcumin films crosslinked with 0.5% citric acid. Gelatin with curcumin films crosslinked with 1% citric acid showed the lowest crystallinity (1.56%). It was concluded that even though citric acid might not prove to be a stable crosslinking agent for the protein (gelatin), it contributed to the antioxidant nature of the films, along with curcumin. These films are promising candidates to be applied on cut fruits, to reduce water loss and oxidation and hence extend their shelf lives.

## 1. Introduction

In the recent past, the food industry all over the world has been seeing revolutionary changes as part of its goal for sustainable development of food and agriculture. A lot of focus is being given to producing packaging materials that help to increase the shelf life of foods while being more environmentally friendly and cost-effective. The use of nanofibers produced from substances like gelatin is being researched to assess the possibility of encapsulating antimicrobial, antibacterial, and antioxidative agents in them to extend the shelf lives of food. To keep foods fresh for longer, a sustainable film must be developed. A lot of factors need to be considered, such as toxicity of the polymer, rheological properties, mechanical strength, gelation temperature, etc.

### 1.1. Use of Gelatin

Gelatin is a polypeptide (protein) derived by hydrolyzing collagen [1] and can be obtained from multiple sources: pigs (porcine gelatin), cattle (bovine gelatin), and the heads, skins, and bones of fish. For packaging materials, bovine gelatin proves to be a logical choice due to its better mechanical properties and biocompatibility. Fish gelatin, although becoming increasingly popular, has the main drawback of being less stable with shear stresses and not having a competitive price due to its recent development [2]. As far as porcine gelatin is concerned, it has the major drawback of being non-halal and non-kosher and hence provides ethical/religious concerns for a large group of consumers, especially in parts of the world such as Asia and Africa. Gelatin from cows has higher gelling and melting temperatures, which makes it a more suitable substance in several food applications [3]. It consists of carboxyl, hydroxyl, and amino groups as functional groups, which also serve as durable film-forming substances [4]. However, the solubility of gelatin needs to be decreased to make it more compatible to be used to produce films for food packaging materials.

### 1.2. Curcumin as Functional Agent

Curcumin, a polyphenol belonging to the family of curcuma (*Curcuma longa* L.), is most commonly found in turmeric and is commonly used to flavor food and also as a dye [5]. Turmeric (curcumin) is also used as an herbal supplement and in the cosmetics industry, as well as the food industry. It has remarkable non-toxic, anti-inflammatory, antioxidant, and antimicrobial properties [6]. In a study conducted by Bojorges et al. [7], the antimicrobial and antioxidant effects of curcumin were discussed, and it was noted that it proved to be better antioxidant in comparison to herbal extracts such as those from seaweed. This proves its ability to preserve food items naturally. However, due to its low water solubility, it is often difficult to incorporate it into foods, packaging, or pharmaceutical products. Of the many solutions to this challenge, one is the method of encapsulation, which produces a delivery system that breaks down gradually with storage time or the effect of temperature [8]. In one study, curcumin was encapsulated in gelatin through electrohydrodynamic atomization, and its antimicrobial and antioxidant properties were assessed [9]. It was seen that the antioxidant nature of the gelatin–curcumin particles was stronger when compared to commercial curcumin powder. The antioxidant property of curcumin is also affected by its extraction method, and it is seen that high heat and pressure extraction proves to be an effective method of extraction and yields improved antioxidant properties, with the concentration of ethanol used for extraction being the most important parameter [10].

### 1.3. Crosslinking with Citric Acid

Crosslinking is a process of joining two or more polymer chains together to obtain a polymer with enhanced properties such as lower solubility and higher thermal stability. One of the commonly known crosslinking agents is glutaraldehyde. It is, however, proven to be toxic to human health, hence it is unadvisable to use it for food packaging. As an alternative, acids such as citric, tannic acid, or ferulic acid may be used. Citric acid is the most cost-effective choice due to its easy availability from citrus fruits and potential compatibility with the polymer solution under study (gelatin/curcumin). Citric acid proves to be biocompatible, non-toxic, and inexpensive, and its carboxyl groups form crosslinks with the hydroxyl groups of the polysaccharide through esterification. Similarly, nucleophilic substitution takes place amongst the carboxyl groups of the acid and amino groups of protein [11]. It has been used as a crosslinking agent in films produced with fish gelatin, but extensive research on its use with bovine gelatin is not available. Porcine gelatin was out of consideration due to its ethical/religious concerns, but the major importance of choosing bovine gelatin, apart from easy availability in comparison to fish gelatin, is its higher proline and hydroxyproline, components of collagens and gelatin necessary for gelation. Total proline plus hydroxyproline values range from 156 residues per thousand to 223 residues per thousand, depending on the source of gelatin (piscine, bovine, or porcine), with fish (cold water and warm water fish) having the lowest amounts, indicating their reason for producing the least stable gelatins [12]. Thus, bovine gelatin proves to have better gelation and hence better physical and rheological properties.

### 1.4. Electrospinning

Electrospinning is a novel method of producing homogenous micro- or nanofibers where the polymer solution is stretched under the effect of an electric field [13]. Some of the biggest benefits of this method are its efficient and simple processing and cheap construction [14]. The use of these fibers is more common in the cosmetics, pharmaceutical, and biomedical industries, but it is an almost uncharted territory in the food industry. It is being explored more now because these fibers have many unique characteristics, including being highly porous and having a larger surface area [15], apart from the ability to hold encapsulated substances to improve bioavailability for functionality [16]. When attached to a plastic sheet, these nanofibers can serve as packaging materials. Gallic acid-loaded electrospun nanofibers were fabricated as active packaging material to protect walnuts from oxidation [13]. These fibers proved to have high thermal stability [17], and the electrospinning method proved to be efficient in encapsulating bioactive compounds (gallic acid). Gelatin–zein–glucose electrospun nanofibers were also produced, with Maillard reaction occurring between the protein and glucose molecules, and these films also showed greater thermal stability and hydrophobic surfaces [18]. In addition to this, ferulic acid was used as an agent to improve the antioxidant nature of fibrous films produced with zein and polyethylene oxide and improved the shelf life of apples. This study showed promising effects of electrospinning on food application [19]. Therefore, electrospun fibers can aid in preventing or delaying oxidation.

### 1.5. Novelty of Study

Gelatin has been successfully doped previously with a formic acid solution to produce nanofibers [20] and has also been used to encapsulate curcumin to widen the potential uses of curcumin in the food industry [9], among other uses. However, to date, there is no study on the interaction of bovine gelatin with curcumin and citric acid. Therefore, the aim of this study was to produce an active packaging film made primarily of gelatin by using electrospinning to extend the shelf life of foods, leading to sustainable development and preservation of the environment. This active film consisted of encapsulated curcumin, which could serve as an antioxidant and antimicrobial agent. Another aim of the study is to apply crosslinking to reduce the solubility of gelatin and to make the polymer solution stronger so that the films obtained would be less susceptible to the environmental conditions. Citric acid was used as the crosslinking agent for this purpose. Moreover, active nanofibers were characterized in terms of morphology, thermal properties, thermogravimetric analysis, antioxidant activity, antimicrobial activity, FTIR, and crystallinity.

## 2. Materials and Methods

### 2.1. Materials

Curcumin (1,7-bis (4-hydroxy-3-methoxyphenyl)-1,6-hepadiene3,5-dione) along with ethanol was purchased from Merck (Darmstadt, Germany). Gelatin was purchased from Eti Gıda Sanayı ve Ticaret A.Ş., Tepebaşı/Eskişehir, Türkiye, while citric acid and acetic acid were purchased from Sigma-Aldrich, Burlington, MA, USA.

### 2.2. Solution Preparation

Gelatin (20%, 25%, 30%, 35%) (*w*/*v*) was put into 40% acetic acid solution and mixed at 900 rpm at 45 °C with a magnetic stirrer until it became homogenous. Thereafter, curcumin was added to ethanol to prepare 0.5% (*w*/*v*) curcumin stock solution. The curcumin stock solution (10 mL) was added to the polymer solutions of gelatin (20 mL) and stirred at 1500 rpm for 30 min at 25 °C. The solution with optimum gelatin concentration, according to the uniformity of fibers as well as their diameters, was crosslinked with citric acid by adding 0.5% and 1% (*w*/*v*) citric acid and homogenizing at 25 °C and 1500 rpm for 10 min.

The nomenclature of the solutions as well as control is given in Table 1, followed by solution and film characterization.

### 2.3. Solution Properties

#### 2.3.1. Rheological Properties

The rheological behaviors of solutions were measured by using a controlled strain rheometer (Brookfield Ametek Coaxial Cylinder RST rheometer, Middleboro, MA, USA) with a coaxial cylinder measurement geometry. The solution was poured into the cylinder and shear stress values were recorded with respect to shear rates varying from 0.013 s^−1^ to 300 s^−1^. The shear stress (*τ*) versus shear rate (*γ*) data fitted well to the power law equation as stated below:

(1) $$\tau =k{\left(\Upsilon \right)}^{n}$$

where *τ* is the shear stress (Pa), *Υ* is the shear rate (s^−1^), *k* is the consistency index (Pa.s^n^), and *n* is the flow behavior index.

#### 2.3.2. Color

The color of curcumin solution was measured by a colorimeter (Konica Minolta CR-5 Osaka, Japan). Results were reported in terms of Hunter L, a, and b values.

#### 2.3.3. Electrical Conductivity

The electrical conductivities of the film-forming solutions (FFSs) containing curcumin and citric acid along with the control were measured using a WTW Inolab Cond 7110 conductometer from Germany at 25 °C. Measurements were carried out twice for each.

### 2.4. Electrospinning

The process of electrospinning was carried out using Nano-Web 103 (Mersin, Türkiye). Each solution was placed in a syringe (5 mL) with a metallic 21-gauge steel needle (11.53 mm inner diameter), placed horizontally on the syringe pump. This was connected to the positively charged electrode that was powered by a direct current (DC) high-voltage supplier. The solution was fed through the metal collector, which was connected to the negatively charged end and covered by aluminum foil at a flow rate of 0.6 mL/h. There was 12 cm between the needle tip and the collector, and the voltage was maintained at 15 kV and humidity at 35% (using silica gels). Experiments were performed at 25 ± 1 °C.

### 2.5. Nanofiber Film Characterization

In order to determine the properties of the nanofibers for comparison, different characterization tests were performed, including SEM imaging, color analysis, antioxidant activity, thermogravimetric analysis (TGA), differential scanning calorimetry (DSC), X-ray diffraction (XRD), water vapor permeability (WVP), FTIR, and antimicrobial analysis, all of which are explained below.

#### 2.5.1. Morphological Analysis

The morphological characteristics of the fibers fabricated were determined using field emission scanning electron microscopy (FESEM) (JEOL, Tokyo, Japan). Samples were stuck on metal stubs and then coated with gold palladium (10 nm). About 50 fibers from each sample were randomly selected from SEM images and their diameters were measured by using ImageJ software V 1.8.0 (NIH, Bethesda, MD, USA).

#### 2.5.2. Color

The color of the films was measured by the same method used for the determination of color of solutions, using a colorimeter (Konica Minolta CR-5 Osaka, Japan).

#### 2.5.3. Antioxidant Activity

The antioxidant activity of the fibers was measured with the 2,2-diphenyl-1-picrylhydrazyl (DPPH) method as stated in Luca et al. [21], but a few modifications were made. The nanofibers (0.05 g) were dissolved in 5 mL of ethanol/water solution (80:20). DPPH radical solution (3.9 mL) with 25 ppm (2.5 mg DPPH/100 mL methanol) was taken and mixed with 100 μL of methanol. One hundred microliters of nanofiber solution was also mixed with 3.9 mL of DPPH radical solution. Absorption of these was measured at 517 nm (A1) by using a UV/Vis spectrophotometer (UV 2450, Shimadzu, Columbia, SC, USA). These were then kept in the dark for 2 h to complete the DPPH solution and curcumin reaction. Then, the absorptions of samples were measured spectrophotometrically again (A2). Methanol was used as a blank. Concentrations (C1 and C2) were found for A1 and A2 using the calibration curve, respectively, and the antioxidant activities (AAs) were calculated according to the equation below [16]:

(2) $$AA\left(mg\frac{DPPH}{g}dry\;weight\right)=\frac{C1-C2}{W_{sample}}\times V$$

where C1 is the concentration of DPPH immediately after the sample (ppm) and DPPH solution are mixed, C2 is the concentration of DPPH 2 h after mixing (ppm), V is the volume of solution (mL), W is the amount of nanofiber (g). After this, the antioxidant activity (%AA) of the electrospun fibers was expressed by the following equation:

(3) $$AA\left(\%\right)=\frac{\left(A_{control}-A_{sample}\right)}{A_{control}}\times 100$$

where A_control_ (A1) and A_sample_ (A2) are the absorbance values of the DPPH solution without and with the presence of the sample solutions.

#### 2.5.4. Thermogravimetric Analyses

TGA 2950 (Exstar TG/DTA 6300, RTI Instruments, Inc., Woodland, CA, USA) was used for thermogravimetric analyses. Roughly 5 mg of nanofiber was heated from 25 °C to 600 °C at a rate of 10 °C/min with nitrogen flowing at a rate of 30 mL/min. Analyses were repeated twice [22].

#### 2.5.5. Differential Scanning Calorimetry

The thermal analysis of the electrospun nanofibers was performed using a differential scanning calorimeter (Pyris 6 DSC, PerkinElmer, Waltham, MA, USA). Approximately 5 mg of sample was put into a hermetically sealed aluminum pan. As a reference, an empty pan was used. After cooling to −50 °C, each pan was heated to 350 °C at a rate of 5 °C/min. The glass transition temperature, melting temperature, and degradation temperature of each sample were determined using differential scanning calorimetry (DSC) thermograms. The DSC measurements were carried out in duplicate [22].

#### 2.5.6. X-ray Diffraction

Crystalline properties of the films were analyzed by an X-ray diffractometer (Rigaku Ultima-IV, USA) using copper (Cu) irradiation with a 30 mA current and 40 kV energy. Samples were scanned at an angular range of 5° and 70° scanning range with 1° min scanning rate. The crystallinity degree of the samples was determined by using the equation below:

(4) $$\mathrm{Total}\;\mathrm{Crystallinity}\;\mathrm{TC}=\frac{Ic}{Ic+Ia}$$

where Ic is the integrated intensity of the crystalline phase and Ia is the integrated intensity of the amorphous phase [23].

#### 2.5.7. Water Vapor Permeability

The water vapor permeability (WVP) of the fibers was determined by the ASTM E96 method. Cups of 40 mm in internal diameter were filled with distilled water in order to create an environment of 100% RH for the nanofibers inside the cup. Nanofibers were stretched over the cups. These cups were then placed into pre-equilibrated desiccator cabinets with a 20% RH and allowed to reach steady state conditions. The cups were weighed every hour. Weight loss versus time was plotted and the water vapor transmission rate was determined from its slope divided by the area of nanofibers that were exposed in the cup. WVP was then calculated using the following equation [22]:

(5) $$WVP=\frac{\left(WVTR)(∆x\right)}{\frac{\left(R1-R2\right)}{100}\times P_{sat}}$$

where WVTR is the water vapor transmission rate (gm^−2^s^−1^), Δx is the thickness of the film (m), R1 and R2 are the relative humidity inside and outside the cups, respectively, and P_sat_ (Pa) is the saturated water vapor pressure at room temperature. Measurements were repeated twice.

#### 2.5.8. Fourier Transform Infrared (FTIR) Analysis

FTIR analyses of electrospun nanofibers were carried out by using an FTIR spectrophotometer (IRAffinity1, Shimadzu, Kyoto, Japan) in attenuated total reflectance (ATR) mode using a diamond ATR crystal. The infrared region analysis was recorded with 40 scans over the wavenumber range of 500–4000 cm^−1^ [17].

#### 2.5.9. Antimicrobial Analysis

The antimicrobial activity of the films was determined by the agar disc diffusion method against *S. aureus* (ATCC 29213) and *E. coli* (BC1402) strains. The isolates were incubated at 37 °C for 24 h in nutrient agar (NA, Merck, Darmstadt, Germany) to obtain early stationary phase cells. The turbidity of the cultures was set to 0.5 McFarland (DEN-1 densitometer; Biosan, Riga, Latvia) (10^8^ cfu/mL). One hundred microliters of the bacterial suspensions was spread on a Mueller–Hinton agar (Condalab, Madrid, Spain) plate. Then, the films were cut into 1 cm diameter discs and placed on the plates in an appropriate manner. The plates were incubated at 37 °C for 24 h. At the end of the incubation, the diameters of the inhibition zones formed around the discs were measured. The tests were performed in triplicate [24].

### 2.6. Statistical Analysis

Analysis of variance (ANOVA) was performed to figure out whether there are noticeable differences between formulations or not, with respect to all separate analyses performed on the solutions and nanofibers (*p* ≤ 0.05). One-way ANOVA was performed and if significant differences were found, a Tukey multiple comparison test was used to compare variables by using MINITAB statistics software (MINITAB for Windows, Version 19, Minitab Inc., State College, PA, USA).

## 3. Results and Discussion

### 3.1. Solution Properties, Fiber Morphology, and Color

The electrical conductivity and rheological properties of solutions were measured to determine their relationship with the fiber morphology, specifically the fiber size diameter (FSD). Certain process parameters as well as ambient conditions were kept constant, such as temperature and RH of the electrospinning environment, voltage applied, and distance between the positive and negative electrodes. The viscosity of a solution helps to determine the nature of the polymer and interactions between its constituents. The electrical conductivity gives an idea of fiber elongation and hence the thickness of the fibers to be obtained. It is noted that a solution with higher electrical conductivity will produce thinner fibers, even spider-web fibers, whereas a solution with lower conductivity will produce thicker fibers [25]. As the electrostatic interactions of a solution increase, its jet elongates more under the effect of an electric field and hence forms thinner fibers. The electrical conductivity depends on different parameters, such as polymer type, solvent type, concentration of polymer, and temperature [26]. In terms of the viscosity of the solutions, if a solution is highly viscous, it will not be able to eject from the spinneret and if it has low viscosity, fibers will not be formed [27]. Therefore, it was important to note the viscosity and its effect on ease of spinning and fiber morphology. Table 2 shows the values of viscosity, electrical conductivity, as well as average fiber diameters obtained from the different solutions and their respective nanofibers.

As can be seen in Table 2, the viscosity of solutions decreased, their electrical conductivity decreased, and their respective nanofiber diameters increased, as curcumin stock solution, and thereafter citric acid, was added to gelatin. A Newtonian characteristic is seen as the n values are close to 1. Apart from that, the viscosity value decreased significantly as the curcumin stock solution was added. This is mainly due to addition of ethanol decreasing the viscosity of the gelatin solution, i.e., diluting it, a pattern that was seen in previous studies as well. When caffeic acid was dissolved in an ethanol–water solution and then added to carob bean–WPC–PEO solution, the solution was seen to become less viscous [28]. Similarly, upon addition of gallic acid to lentil flour-based nanofiber solutions, it was observed that the consistency index (k) values decreased, which is an indication of decreasing viscosity [13]. Furthermore, viscosity was also seen to increase with increasing gelatin concentration from 7 to 20% (*w*/*v*) [26]. From this, it can be inferred that the decreasing viscosity in our solution was expected as the overall concentration of gelatin in the solutions decreased when curcumin mixed with ethanol was added and decreased further upon addition of citric acid.

**Figure 1:**
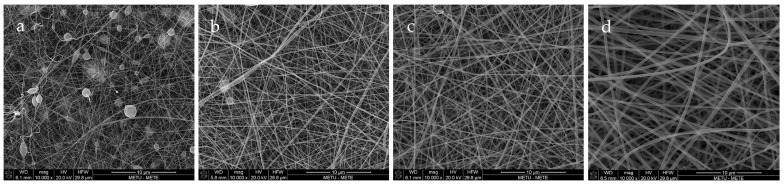
(**a**) The 20% GL, (**b**) 25% GL, (**c**) 30% GL, (**d**) 35% GL nanofiber morphology.

Figure 1 shows the fiber morphologies through SEM images of fibers with different gelatin concentrations, varying from 20–35%. This experiment was performed to determine the optimum gelatin concentration by considering the size and uniformity of fibers. As can be seen, the 20% and 25% GL nanofibers were non-uniform and contained beads, both of which were unacceptable attributes in electrospun fibers. Hence, due to the uniformity and neatness of 30% and 35% GL, the study was narrowed down to these concentrations. Thus, curcumin solution was added to both and based on the nanofibers with a lower diameter (182.246 nm for 30% GLC and 263.754 nm for 35% GLC), the 30% GL with curcumin stock solution added was chosen before adding citric acid.

**Figure 2:**
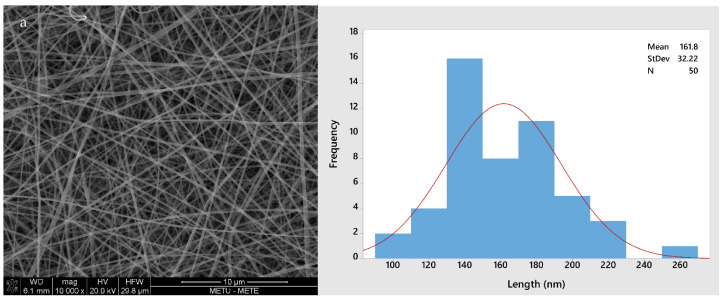

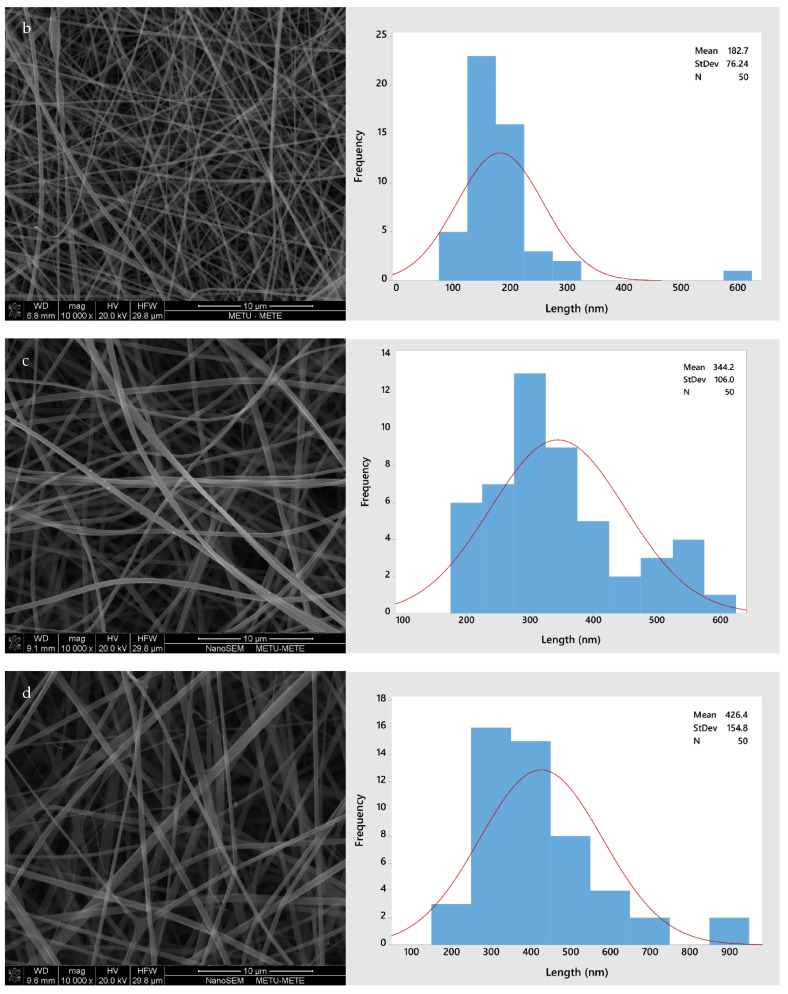
SEM images and fiber size distributions with normal curve (red): (**a**) GL; (**b**) GLC; (**c**) GLCCA0.5; (**d**) GLCCA1.

Figure 2 shows the morphology of all four different fiber types (GL, GLC, GLCCA0.5, GLCCA1). The diameters increased from GL to GLCCA1 (161.8 nm to 426.4 nm). It is known that the diameters of electrospun fibers are highly dependent on the electrical conductivities of their respective solutions, with a higher electrical conductivity leading to smaller fiber diameters, a case seen in this study as well (Table 2). This is due to higher electrostatic forces and hence longer jet elongation under the voltage applied in the electrospinning machine, leading to thinner diameters. Surface tangential stress is created when charge is more easily accumulated in a solution due to higher conductivity, which aids in fiber elongation [29]. Solutions with zero electrical conductivity cannot produce fibers, whereas if the conductivity is too high, electrical discharge into the surroundings occurs. It was seen that when the conductivity of PEO solution was increased to 0.5 S m^−1^ (5 mScm^−1^), the stable cone jet changed to a multi-jet expulsion from the needle [30] and the solution could not be spun. Previously, gelatin was doped with formic acid by dissolving gelatin solution in formic acid solution to obtain improved properties, such as enhanced stability, in the nanofibers [20]. The fiber diameters increased upon addition of formic acid, and this was parallel to the diameters increasing with citric acid addition for crosslinking. 

Table 3 shows the L, a, and b values of the solutions and their corresponding films, measured according to the Hunter Lab scale. Focus was drawn towards the lightness (L*) and yellowness (b*) of the solution and films. The solution became lighter upon addition of citric acid, in comparison to solutions with only gelatin and curcumin (GLC), owing to the pH sensitivity of curcumin and protein structure of gelatin. A drastic value change was seen in the b values (blue–yellow scale), where both the solution and films became more yellow upon addition of curcumin, an expected change due to the rich yellow pigment of curcumin.

### 3.2. Antioxidant Activity

Curcumin is known to scavenge free radicals and hence its antioxidant activity was determined. It is known that the antioxidant activity of curcumin originates from its phenolic hydroxyl group and ethylene group of β-diketone moiety [31]. As can be seen in Table 4, it was found that the antioxidant activity of the nanofiber films decreased significantly upon addition of citric acid for crosslinking and thereafter increased significantly as well upon addition of a higher percentage of citric acid. It is also known that the carboxyl groups of citric acid react with the active sites of curcumin [22], a reaction that may lead to a reduction in the number of active sites available for the antioxidant activity of curcumin. This decrease in AA is also attributed to crosslinking, as the release rate is expected to be lower in a crosslinked polymer matrix and hence its resulting films have relatively lower antioxidant activity [32]. Contrary to this finding, ineffective encapsulation of curcumin and crosslinking in the film can also be inferred from the fact that the antioxidant activity of the films (due to curcumin) did not increase in the presence of citric acid [33] and, in fact, decreased by almost 30%. However, upon addition of a higher amount of the crosslinking agent, a slight increase in AA was seen from GLCCA0.5 to GLCCA1, by another 24%. This is attributed to AA resulting from the additional citric acid, not from curcumin itself [34].

### 3.3. Thermogravimetric Analysis (TGA)

**Figure 3:**
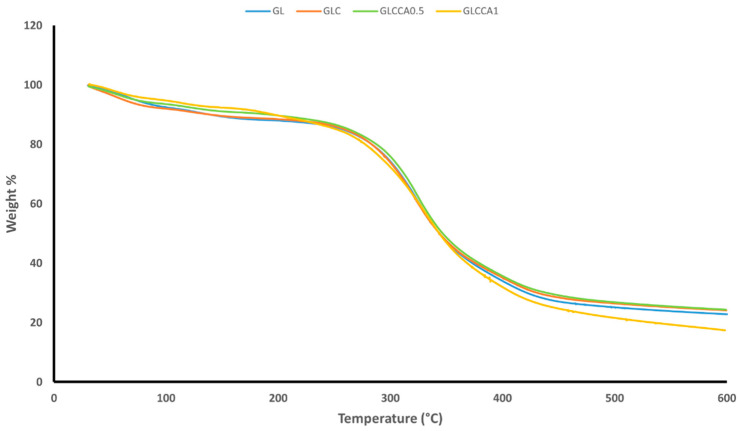
TGA weight loss graph.

The thermogravimetric analysis was performed to understand the weight loss variation of the films with increasing temperature. TGA gives an idea of certain thermal events that occur on films, such as absorption, adsorption, vaporization, decomposition, etc. [35]. Figure 3 shows the weight loss versus temperature graphs of the nanofiber films, along with Table 5 showing the weight loss percentages. It can be noted that the films follow a two-step weight loss, with the first one being responsible for evaporation of water and other volatile compounds, centered around 100–150 °C [23]. The second stage of weight loss is due to evaporation of citric acid derivatives in the film [36], followed by decomposition linked to depolymerization reactions [37] and covalent bond cleavage [38]. The peak of the second stage is centered around 400 °C. Upon addition of curcumin and further upon addition of the crosslinking agent, citric acid, the weight losses are seen to decrease slightly, indicating the occurrence of crosslinking and strong intermolecular interactions between the components of the polymer solution.

### 3.4. Differential Scanning Calorimetry (DSC)

This thermo-analytical technique provided information on the difference in energy and heat required to increase the temperature in the samples. The glass transition temperature, Tg, shown by a shift in the graph, was seen between 68 °C and 120 °C, related to the glass-to-rubber transition of amino acid blocks in the peptide chain [39]. This was followed by the melting temperature, Tm, indicated by an endothermic peak, between 70 °C and 80 °C. Tg is the softening point of the films and Tm represents the dissociation of the ordered regions or melting point of the crystal phase of the material. Table 6 shows the Tg, Tm (along with onset and end temperatures), as well as degradation temperatures of the films. The films show a semi-crystalline structure due to the presence of both Tg and Tm, and the fibers mostly remain in a non-crystalline state as a result of elongating and rapidly solidifying during electrospinning [23]. GL films melt at a similar temperature to that studied in bovine gelatin films earlier [39] but show a higher Tg than gelatin films produced through casting [40]. An increase in Tg is seen upon addition of curcumin in gelatin and this may be due to the curcumin causing inflexibility in the amorphous polymer chain movement, higher hydrogen bonding, and stronger crystalline forces [41]. The films were also seen to become less sticky, which is an expected observation upon an increase in Tg. Further, upon addition of citric acid, the Tg was seen to decrease in comparison to GLC. The most significant reason may be citric acid acting as a plasticizer and increasing the free volume between the polymer chains, but since this Tg is greater than the Tg for GL, it may be an indication of crosslinking [41]. An overall decrease in melting temperature was seen as curcumin and citric acid were added, associated with the strengthening of the polymer solution with hydrogen bonding. By looking at the degradation temperature data, an increase in the amorphous nature of the polymer solution due to crosslinking was noticed in gelatin fibers prepared from gelatin–formic acid solution, wherein there was a higher amount of random coil conformation and lower helical conformation [20]. Even though an overall decrease in degradation temperature was also seen upon addition of the crosslinking agent in the films in this study, this decrease was not significant and therefore a noteworthy change in conformation could not be concluded.

### 3.5. Crystallinity Analysis (XRD)

**Figure 4:**
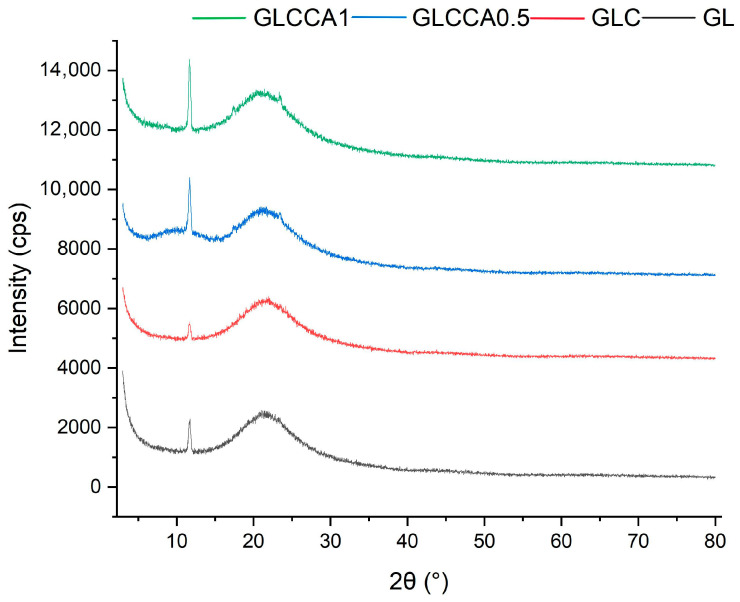
XRD graphs of films.

To understand the change in crystallinity of the gelatin films with respect to curcumin solution and citric acid, the XRD analysis was performed, and its resulting graphs are shown in Figure 4. As shown in Figure 4, the crystallinity is generally quite low for the films and an overall decreasing trend can be seen. In a previous study [20], it was stated that the crystalline structure of gelatin results from its α-helix and triple helical structure and that the crystallinity of aqueous gelatin solutions is lower, with more amorphous structures seen in nanofibers (based on a gelatin and formic acid mixture). Similarly, crystallinity of GL decreases in GLC and further in GLCCA because the carboxyl groups of CA react with the hydroxyl and amine groups of the protein, causing hindrance in the formation of crystal structure [23]. The crystallinity is also hindered due to the NH_2_ groups in gelatin favoring hydrogen bond formation in the polymer chain, further strengthening the films [42].

### 3.6. Water Vapor Permeability

The main function of testing water vapor permeability is to determine how strong the water barrier in a packaging film is between the food product being packaged and the surroundings. Water vapor permeability (WVP) of the films is aimed to be as low as possible. It is generally seen for hydrophilic films, such as gelatin, that the WVP increases with increasing thickness of films. Even though the films in this study were very thin (~0.05–0.08 mm), the nanofibers provided a good barrier for water and there was no significant difference seen in WVP. Overall, it can be said that the WVP was quite low, as shown in Table 7. In a study conducted to compare the WVP of fish gelatin with that of mammalian gelatin (bovine and porcine), mammalian gelatin was found to have a higher WVP due to higher amounts of proline and hydroxyproline in its structure, causing it to have a higher affinity for moisture [43]. The WVP of gelatin films was higher than that seen in Table 7, which was attributed to the solvent used (water) for dissolution as well as the casting method used instead of electrospinning. Similar to our results, starch–fish gelatin films also did not show a significant difference in their WVP when the starch–gelatin ratios were changed and plasticized with glycerol [26]. WVP depends on many factors, including the ratio between crystalline and amorphous zones, interactions between functional groups, etc. [44], but the slight difference seen could be due to difference in water molecule diffusion (through the nanofiber matrix), hydrophilic–hydrophobic ratio [45], and viscosity of film-forming solutions. The drop from 4.395 × 10^−10^ to 3.590 × 10^−10^ could be linked to the hydrophobic nature of curcumin, which might have a decreasing effect on water solubility [46]. Upon addition of citric acid, the WVP is seen to be higher than that of fava bean–chitosan–curcumin films crosslinked with citric acid, since citric acid is known to be better at forming hydrophobic ester bonds with polysaccharides than with proteins [23].

### 3.7. Fourier Transform Infrared (FTIR) Analysis

**Figure 5:**
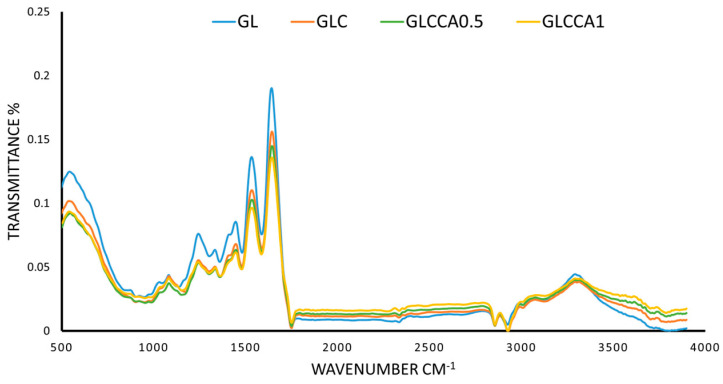
FTIR spectra of films.

The FTIR analysis was conducted in order to identify characteristic peaks and functional groups present in the thin films. The characteristic amide peaks (Amide I, Amide II, Amide III and Amide A) of gelatin were seen in all four films with slight differences in wavenumbers, as shown in Figure 5. The peak at 1640 cm^−1^ (Amide I) corresponds to the C=O stretching vibration of gelatin, linked to the random coil and α-helix conformation of gelatin [47,48]. At 1530 cm^−1^ (Amide II) and 1240 cm^−1^ (Amide A) the peaks correspond to bending of N-H and stretching of C-N, respectively [47], while N-H stretching is seen from 3100–3500 cm^−1^, with a peak at 3290 cm^−1^. No functional groups are altered upon addition of substances to the polymer solution, verified by the fact that all characteristic peaks of GL are present in the rest of the films as well [49]. However, as a change in intensity of the peaks can be seen, with the largest difference seen in GLCCA1 for all peaks, it can be concluded that the helix or protein secondary structure has been altered [39] and therefore some crosslinking has occurred.

### 3.8. Antimicrobial Activity

**Figure 6:**
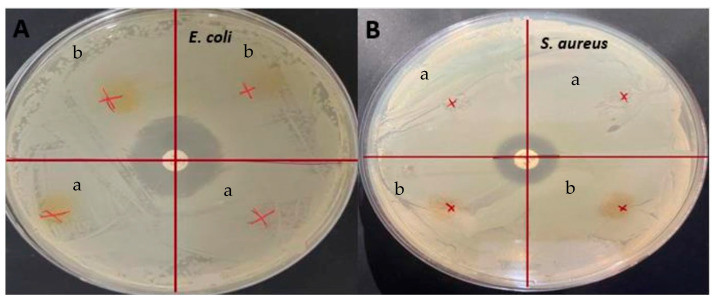
Antimicrobial analysis: (**A**) *E. coli* (**B**) *S. aureus* (a) GLCCA0.5; (b) GLCCA1.

Antimicrobial tests were conducted on the films and the presence of inhibition zones was investigated, against *E. coli* and *S. aureus*, as shown in Figure 6. Even though these films showed significant antioxidant activity, no inhibition zones were seen on any of the films. Similarly, it was determined that although the gelatin/curcumin-based films had significant antioxidant properties, they did not exhibit antimicrobial activity against *E. coli* and *S. aureus* [50]. Since the antimicrobial activity was largely dependent on curcumin with GL being used as the control film, the main reason for no inhibition in the present study could be the low concentration of curcumin. Similar results were obtained previously when sumac was incorporated into fava bean flour active films [51]. There was no antimicrobial activity detected with 5% sumac, for *S. aureus*, but it increased significantly when the sumac amount was increased to 10% and 20%. In this regard, it has been reported that the minimum concentration required for aqueous extraction of *C. longa* to show antimicrobial activity against *E. coli* and *S. aureus* was 4–32 g/L [52]. In another study, it was stated that the minimum inhibitory concentration value of ethanolic turmeric extract with *S. aureus* was 31.25 ppt [53]. In addition, one of the causes of curcumin’s antimicrobial activity is its ability to alter membrane permeability and inhibit bacterial growth [54]. Hydrogen bonding and hydrophobic interactions of bacterial cell phenols and membrane proteins are responsible for this effect of curcumin on bacteria. However, in protein-based films, proteins can interact with phenolic compounds such as curcumin, resulting in blocking the active sites desired for antimicrobial activity [55]. It was found earlier that protein films loaded with phenolic compounds did not provide antimicrobial activity, and it was reported that this result may be related to the interactions of phenolic compounds with proteins and the alkaline pH of the film dispersions [56]. Apart from that, it was found that nanocurcumin had a larger inhibition zone than bulk curcumin and was a more effective antimicrobial agent due to the reduced particle size [57]. In light of the literature data, it is thought that the lack of antimicrobial activity of the films obtained in the present study may be due to the nature of curcumin, the low concentration of curcumin, and possible interactions between gelatin and curcumin.

## 4. Conclusions

This study was aimed at employing the method of electrospinning, with bovine gelatin as the base, curcumin added as a functional agent, and citric acid used for crosslinking, to produce a packaging film with enhanced antioxidant activities. It was seen upon addition of curcumin and further upon addition of citric acid that the diameter of the fibers increased significantly but uniform fibers were still obtained. The antioxidant activity of GLC was seen to be the highest, and its weight loss and crystallinity data were seen to be promising for it to be used as a packaging film. In GLCCA0.5 and GLCCA1, a few properties, such as antioxidant activity, thermal properties, and WVP, were enhanced while others were reduced or unchanged, such as film Tdeg. Between 0.5% and 1% citric acid addition, the 1% citric acid crosslinked films were the winning candidate with respect to improved amorphous nature (decreased crystallinity) and free radical scavenging (antioxidant nature), while GLCCA0.5 was better at being thermally stable by showing the lowest weight loss when heated and highest melting temperature and providing the strongest barrier against water. Therefore, curcumin and citric acid paired with gelatin are a promising combination to be used as a packaging material for cut fruits.

## Figures and Tables

**Table 1:** Nomenclature of solutions and their respective films.

Solution/Film Name	Contents
GL	30% gelatin (*w*/*v*) solution
GLC	30% GL (*w*/*v*) solution mixed with 0.5% curcumin (*w*/*v*) in ethanol
GLCCA0.5	30% GL (*w*/*v*) solution mixed with 0.5% curcumin (*w*/*v*) in ethanol and 0.5% CA (*w*/*v*)
GLCCA1	30% GL (*w*/*v*) solution mixed with 0.5% curcumin (*w*/*v*) in ethanol and 1% CA (*w*/*v*)

**Table 2:** Solution properties of polymer solutions and respective fiber diameters.

Solution/Film	Viscosity (Pa.s)	Electrical Conductivity (mS/cm)	FSD (nm)
GL	0.41433 ± 0.0083 ^a^	2.74533 ± 0.0019 ^a^	162.420 ± 0.916 ^d^
GLC	0.16786 ± 0.0117 ^b^	1.30757 ± 0.0032 ^b^	182.246 ± 0.619 ^c^
GLCCA0.5	0.14931 ± 0.0102 ^b^	1.29360 ± 0.0048 ^b^	344.033 ± 0.304 ^b^
GLCCA1	0.14040 ± 0.0165 ^b^	1.17300 ± 0.0093 ^c^	427.417 ± 1.390 ^a^

Columns with different letters are significantly different to each other (*p* < 0.05). FSD: Fiber Size Diameter.

**Table 3:** Colorimetry results (L*, a*, and b* values).

Type		L*	a*	b*
GL	Solution	90.6350 ± 0.0071 ^a^	−3.980 ± 0.2260 ^a^	17.330 ± 0.325 ^c^
	Film	19.255 ± 0.2900 ^b^	−1.190 ± 0.141 ^c^	1.200 ±0.226 ^c^
GLC	Solution	82.0350 ± 0.0778 ^c^	4.350 ± 0.0283 ^c^	56.865 ± 0.0495 ^b^
	Film	13.915 ± 0.2620 ^c^	5.330 ± 0.382 ^a^	8.345 ± 0.304 ^b^
GLCCA0.5	Solution	83.120 ± 0.1980 ^b^	2.430 ± 0.0283 ^b^	57.790 ± 0.156 ^ab^
	Film	20.605 ± 0.4030 ^a^	3.350 ± 0.382 ^b^	13.170 ± 0.339 ^a^
GLCCA1	Solution	83.4800 ± 0.1273 ^b^	2.205 ± 0.191 ^b^	57.990 ± 0.311 ^a^
	Film	14.540 ± 0.0424 ^c^	3.845 ± 0.191 ^b^	9.325 ± 0.460 ^b^

Columns with different letters are significantly different to each other (*p* < 0.05).

**Table 4:** Antioxidant Activity of films.

Film	AA (%)
GLC	32.01 ± 1.88 ^a^
GLCCA0.5	22.47 ± 0.63 ^b^
GLCCA1	27.93 ± 0.78 ^a^

Columns with different letters are significantly different to each other (*p* < 0.05). AA: Antioxidant Activity.

**Table 5:** Two-stage weight loss percentage and crystallinity percentage of films.

Film	Weight Loss 1 (%)	Weight Loss 2 (%)	Crystallinity (%)
GL	11.110 ± 0.4330 ^a^	61.430 ± 0.2520 ^b^	2.220 ± 0.2520 ^b^
GLC	10.200 ± 1.7140 ^ab^	60.960 ± 0.1153 ^b^	1.626 ± 0.3150 ^b^
GLCCA0.5	8.763 ± 0.9120 ^ab^	60.907 ± 0.5550 ^b^	6.50 ± 1.5400 ^a^
GLCCA1	8.047 ± 0.5530 ^b^	63.180 ± 0.9430 ^a^	1.5625 ± 0.0898 ^b^

Columns with different letters are significantly different to each other (*p* < 0.05).

**Table 6:** Glass transition (Tg), Melting (Tm), and Degradation Temperatures (Tdeg) of nanofiber films.

Film	Tg (°C)	Tm Start (°C)	Tm Peak (°C)	Tm End (°C)	Tdeg (°C)
GL	69.16 ± 1.460 ^c^	55.10 ± 1.57 ^a^	80.285 ± 0.728 ^a^	106.36 ± 4.40 ^b^	231.515 ± 1.068 ^a^
GLC	119.730 ± 1.117 ^a^	48.98 ± 2.93 ^a^	73.835 ± 1.308 ^c^	98.52 ± 8.79 ^b^	227.745 ± 1.280 ^a^
GLCCA0.5	112.73 ± 1.640 ^b^	32.35 ± 2.80 ^b^	78.055 ± 1.407 ^ab^	142.69 ± 3.20 ^a^	230.140 ± 2.570 ^a^
GLCCA1	110.49 ± 1.440 ^b^	46.56 ± 2.70 ^a^	76.010 ± 0.170 ^bc^	103.25 ± 3.14 ^b^	226.050 ± 1.004 ^a^

Columns with different letters are significantly different to each other (*p* < 0.05). Tg: Glass Transition Temperature; Tm: Melting Temperature.

**Table 7:** Water Vapor Permeability of films.

Film	WVP (×10^−10^) gm^−1^ s^−1^ Pa^−1^ Unit	Thickness (mm)
GL	4.395 ± 0.00 ^a^	0.08763 ± 0.00348 ^a^
GLC	3.590 ± 0.00 ^bc^	0.05858 ± 0.00813 ^b^
GLCCA0.5	2.365 ± 0.00 ^c^	0.04950 ± 0.00337 ^b^
GLCCA1	4.695 ± 0.00 ^ab^	0.08225 ± 0.00271 ^a^

Columns with different letters are significantly different to each other (*p* < 0.05).

## Data Availability

The data presented in this article are available on reasonable request from the corresponding author.
